# End of life care interventions for people with dementia in care homes: addressing uncertainty within a framework for service delivery and evaluation

**DOI:** 10.1186/s12904-015-0040-0

**Published:** 2015-09-17

**Authors:** Claire Goodman, Katherine Froggatt, Sarah Amador, Elspeth Mathie, Andrea Mayrhofer

**Affiliations:** Centre for Research in Primary and Community Care University of Hertfordshire, Hatfield, AL10 9AB UK; International Observatory on End of Life Care University of Lancaster, Lancaster, UK; Marie Curie Palliative Care Research Department University College London, London, UK; Centre for Research in Primary and Community Care University of Hertfordshire, Hatfield, UK; Centre for Research in Primary and Community Care University of Hertfordshire, Hatfield, UK

## Abstract

**Background:**

There has been an increase in research on improving end of life (EoL) care for older people with dementia in care homes. Findings consistently demonstrate improvements in practitioner confidence and knowledge, but comparisons are either with usual care or not made. This paper draws on findings from three studies to develop a framework for understanding the essential dimensions of end of life care delivery in long-term care settings for people with dementia.

**Methods:**

The data from three studies on EoL care in care homes: (i) EVIDEM EoL, (ii) EPOCH, and (iii) TTT EoL were used to inform the development of the framework. All used mixed method designs and two had an intervention designed to improve how care home staff provided end of life care. The EVIDEM EoL and EPOCH studies tracked the care of older people in care homes over a period of 12 months. The TTT study collected resource use data of care home residents for three months, and surveyed decedents' notes for ten months,

**Results:**

Across the three studies, 29 care homes, 528 residents, 205 care home staff, and 44 visiting health care professionals participated. Analysis of showed that end of life interventions for people with dementia were characterised by uncertainty in three key areas; what treatment is the 'right' treatment, who should do what and when, and in which setting EoL care should be delivered and by whom? These uncertainties are conceptualised as Treatment uncertainty, Relational uncertainty and Service uncertainty. This paper proposes an emergent framework to inform the development and evaluation of EoL care interventions in care homes.

**Conclusion:**

For people with dementia living and dying in care homes, EoL interventions need to provide strategies that can accommodate or "hold" the inevitable and often unresolvable uncertainties of providing and receiving care in these settings.

## Background

Although the prevalence of people with dementia in the community has not increased at the rate projected by research in the nineties, the numbers of people with dementia in long term care settings have. In the UK, approximately 460 000 people live in care home settings. Two thirds or more of residents have dementia and are in the last years of life [1–3]. Care home residents are a population who can no longer be supported to live independently at home [4]. For the majority of care home residents the anticipation of dying is not the reason for admission even though they are in the last years of life and consequently the dying experience is highly variable [5–8].

Care homes in the UK may be categorised into residential care homes that provide personal care only and nursing homes that provide personal and nursing care. Residents’ medical care and support from specialist palliative nursing and therapy services are provided by visiting health care professionals. It is increasingly recognised that care homes are important providers of palliative care for older people and the issues faced by UK care homes are similar to those in Europe [9].

### Care provision

Research, training, and dementia sensitive tools to improve end of life care for people in long term care settings have focused on evidence of anticipatory care, the use of relevant assessment tools, reduction in symptoms, cost effectiveness, place of death and related measures of quality [6, 10]. However, care provision and decision making in relation to EoL care for care home residents is often complicated by the multiple, sometimes transient care staff, professionals, and resident representatives involved [11]. As many as 27 different health care services can visit to provide care and treatment for residents in care homes [12]. This is compounded by a variably qualified workforce with inconsistent access to clinician support, different regulatory approaches for long-term care providers, and differing systems of service delivery, coverage and reimbursement [13].

### Evaluating care provision

Evaluations of EoL care for people with dementia in long term care settings focus on documentary evidence of anticipatory care as a proxy for EoL care planning, staff satisfaction/ confidence, the use of relevant assessment tools, reduction in symptoms, cost effectiveness, and place of death (e.g. [14–16]). Usually, the comparison of outcomes is between the end of life intervention and “usual care”. It is therefore perhaps unsurprising that interventions that provide extra support, structured approaches to care and additional training and resources achieve better outcomes.

There is a need to differentiate between the relative effectiveness and suitability of different end of life interventions developed for care home populations, and specifically, those with dementia. In this paper, we offer a framework for the development and evaluation of EoL care interventions for people with dementia in care homes, and argue that key to an intervention’s effectiveness is its ability to ameliorate and hold the uncertainty inherent to end-of-life care in dementia.

## Method

The conceptual framework proposed in this paper is based on the secondary analysis of data from three recently completed standalone studies, all of which were specifically concerned with end of life care for older people in English care homes (i) EPOCH (ii) EVIDEM EoL, and (iii) TTT EoL.

### Overview of the original studies and data collection procedures

#### The EPOCH study (2008–2010)

The Experiences of Older People of living and dying in a Care Home (EPOCH) study aimed to understand how older people discuss and experience living and dying in care home over time. The study used a prospective mixed methods approach to understand how living in a care home influences residents’ views, experiences and expectations of end-of-life care including symptom relief, and establish how care home staff and visiting health practitioners’ understand and interpret their roles and responsibilities in EoL care. It tracked the events and care received by 121 residents in six residential (without onsite nursing) care homes in the East of England over 12 months, through review of care home notes at four time points (and post-death analyses where applicable) as well as semi-structured interviews with 63 care home residents, each of whom were interviewed up to three times within the time period. These data were complemented by semi-structured interviews with 30 care home staff and 19 visiting health care professionals. A more detailed account of the recruitment, methods and findings from this study is provided elsewhere [17–20].

#### The EVIDEM EoL Study (2009–2011)

The EVIDEM End of Life (EVIDEM EoL) study investigated the dying trajectories of older people living in care homes with a focus on residents with dementia specifically, and also went on to develop an intervention to improve end-of-life care for residents with dementia in these settings. Phase One of the study used –as in the EPOCH study—as prospective mixed methods approach to establish resident characteristics, key events, services and resources used, and care home staff and visiting health practitioners’ understanding and interpretation of their roles and responsibilities in EoL care. It tracked the events and care experienced by 133 older people with dementia living in another six residential (without onsite nursing) care homes also in the East of England over 12 months, through review of residents’ care notes at three different points in time and, where applicable, post-death. These data were complemented by semi-structured interviews with 18 people with dementia, 33 care home staff and 14 visiting health care professionals. Significance testing of distribution of baseline characteristics used Wilcoxon rank-sum test for continuous variables and chi-squared test for categorical variables. A more detailed account of the recruitment, methods and findings from the EVIDEM EoL study is provided elsewhere [10, 15, 20–25].

#### The TTT study (2012–2013)

The Train the Trainer in End of Life care (TTT EoL) study evaluated the effectiveness of a peer-to-peer EoL care education and training programme in relation to the intervention’s ability to reduce treatment, relational and service uncertainty. The programme was implemented in 17 care homes and collected resident characteristics data and resource use data from a randomly selected sample of 274 residents (30 % of the population) across three sites for a period of 12 weeks. Sixty nine percent of residents staying in residential care homes, and 64 % of residents staying in care homes with nursing on site had a diagnosis of dementia. Data relating to advance care planning (ACP) and dying trajectories were collected via a survey of 150 care notes of residents who had died in 12 of the participating care homes over a period of ten months. Qualitative data were collected from trainers, programme learners, care home managers, and clinical palliative and EoL care specialists via interviews, observation of workshops, focus groups and audio diaries. Details of methods and results are reported elsewhere [26].

### Secondary data analysis

A series of meetings convened by CG over 2012–2013 was held with all co-authors to undertake additional in-depth analysis of findings from the primary studies described above. All were part of the original research teams on at least one of the above mentioned studies, and were involved in all stages of data collection, analysis and dissemination of results. CG led all three studies. EM and KF were part of the on the EPOCH original research teams EM and SA worked on EVIDEM EoL, and AM and SA on the TTT study. This meant that meetings could draw on the authors’ detailed familiarity with the data, the databases and understanding of the three studies’ questions and most importantly, the care home contexts and how data was co-constructed between participants and researchers [27]. All authors were invited to examine and discuss primary data (i.e. resource use and interview data) from all three studies, and specifically how these mapped onto the emerging framework conceptualised by CG. Authors undertook a deductive thematic analysis directed by the concept of uncertainty in end-of-life care in dementia, through debate on the similarities and differences in primary data across studies to further specify and refine the different categories of uncertainty until a consensus was achieved.

## Ethics

All three studies were approved by national health and social care research ethics committees and health and social care governance permissions were secured through the relevant NHS and Local Authorities. EPOCH (REC reference 08/H0502/38); Evidem EoL (REC reference: 08/H0502/74) Train the Trainer (REC 12/WA/0384).

Written informed consent was obtained for all participants’ interviews and for the review of care home notes. Where residents lacked capacity to consent to their notes being reviewed, their personal consultees were asked to judge if they thought the study would have been of interest to the resident prior to their loss of capacity.

## Results

The EVIDEM EoL study had recorded 27 deaths (20 %) across the six care homes, and the EPOCH study 23 deaths (19 %) both, over a period of 12 months. The TTT EoL study accessed decedents’ notes of 150 residents who had died over ten months, since the start of the TTT intervention across 12 care homes

When decedents’ dying trajectories in the EVIDEM EOL and EPOCH studies were compared with the illness trajectories of residents who were still alive, there was nothing to differentiate between the two groups in terms of resident characteristics, episodes of ill health, or key events. For the majority of care home residents who had died there was an identifiable period when they were approaching the end of life and planned care was put in place. This was also the case in the TTT EoL study, where 118 of 150 deaths had been expected. However, the recognition that someone may be dying did not preclude the experience of uncertainty leading up to that point, in terms of deciding how the dying process was to be managed, discussed and resourced. There were three ways in which uncertainty was experienced: (i) treatment uncertainty refers to recognising whether someone was actively dying, to decision-making about treatments, referrals to hospital, and how the goals of care were informed by residents’, staff and families’ different definitions of quality of life, (ii) relational uncertainty related to how EoL care decisions are shaped by relationships and roles within the care home, with visiting health care professionals, and by relationships with external regulatory bodies, and finally and finally (iii) service uncertainty refers to how the organisation and delivery of services can influence decisions about place of care, resource allocation and continuity of care.

## Treatment uncertainty

Residents in all three studies had limited life expectancy due to their advanced age and to co-morbidities. For residents who were unwell but not close to death, treatment uncertainty arose when the presence of dementia complicated assessment. Treatment uncertainty was evident in three situations, (i) when a resident had been stable with no signs of decline, (ii) when a resident had previously recovered from a similar episode of ill health, for example a urinary tract infection, and (iii) when the period of deterioration was protracted with weeks and sometimes months of good health between episodes of ill health. Treatment uncertainty was further complicated by how quality of life was defined, and whether interventions were desirable. For care home staff, residents and family members quality of life was linked to residents’ ability to respond, engage with others and to appreciate the home environment. People with dementia who were able to take part in an interview described that what was most important to them was the ability to interact with staff, see family, and feel that they were valued and safe. For visiting primary care staff quality of life was linked to what they would want for themselves, and whether the intervention would lead to recovery and/or to functional improvement.

The competing narratives of what a good outcome looked like when someone was ill or vaguely unwell could not be resolved by reference to a person’s advance care plan (ACP), which in the main referenced key transition points. In two of the studies (EPOCH and TTT EoL) the majority of ACPs had been completed only at the point when it was clear to all involved that the person was actively dying, which was in the last few days or, in one case, the last few hours of life (Table 1).

**Table 1:** Treatment uncertainty

**Treatment uncertainty** refers to recognising whether someone was actively dying, to decision-making about treatments, referrals to hospital, and how the goals of care were informed by residents’, staff, family’ different definitions of quality of life.
**Care home manager talking about the difficulties of knowing if a resident is approaching the end of life:**
*“I don’t think you could say there is a usual pattern (…) Eating and drinking] can be the start of something…it can be, that’s what I mean. It doesn’t mean to say that that’s going to be* Tender Loving Care *it could just mean that they’re a bit unwell at that particular time”* (EVIDEM EoL).
**Treatment uncertainty where GP does not know at what point he should be taking lead in decision making:**
*“In an ideal world it would be a GP [deciding that an older person with dementia requires EOL care]. Everybody making that decision ….. because it’s undefined isn’t it? An undefined period of death…* (EVIDEM EoL).
**This resident questioned the value of advance care planning as she saw that it was impossible to know how and when she would die:**
*“I really don’t see that there is an obligation to foresee all the circumstances that might happen to a person, that might make it very difficult when you come to die, that everything will be ready but it isn’t now and it never will be, whatever you supply*” (EPOCH).
**Comment by care home staff member referring to treatment uncertainty after completing EoL training:**
*“…when residents are getting better we always used to think ‘that’s it, they are getting better’… but it’s not. And it must be an emotional roller-coaster for a relative to hear that their parent has gotten worse, then better, then worse….*”(TTT study)*.*

## Relational uncertainty

Relational uncertainty operated at an interpersonal and organisational level. At an interpersonal level there was uncertainty about the roles and responsibilities of care home staff, family members and primary care staff when a resident was dying. Although decisions to treat were GP led, this was a complex three way process between care home staff, visiting health care professionals and family. Visiting GPs had the (clinical) authority to make key decisions about hospitalisation or treatments, but this was undermined by their infrequent contact with, and partial knowledge of, the older person with dementia. For health care professionals who visited to provide urgent or emergency care, navigating these relationships was even more problematic.

The effectiveness of this process was predicated on existing relationships, known patterns of working and the number of people involved. General practitioners (GPs) and district nurses relied on care home staff to provide them with information about the resident, to help them interpret what ‘normal’ looked like for the person with dementia, alert them to deterioration, and act as a conduit for information to and from family members. Similarly, care home staff often relied on family members to know the older person’s story and preferences. There were systems to support this process, such as the use of shared documentation, advance care plans and, in a few cases, palliative care registers. However, paperwork alone did not initiate or guide conversations (see Table 2). Everyday relationships had to be sufficiently robust to allow debates around decisions, conversations with relatives, and discuss treatment options, on an ongoing basis. The EVIDEM EoL study, for example, found a positive association between a resident having been admitted from a relative’s home and a reduced risk of unplanned hospital admission.

**Table 2:** Relational uncertainty

**Relational uncertainty** relates to how EoL care decisions are shaped by relationships, responsibilities and roles within the care home, with visiting health care professionals, and by relationships with external regulatory bodies.
**Example of how the relationship between paramedics and care home staff can be affected by different expectations:**
*“… you have [emergency service] staff. they would think ‘Well, this patient needs to be in hospital for whatever reason’, and really you might get a conflict, but it depends on the way that the communication is going between the two. It’s quite often key to the decisions that are made as well, and sometimes it starts to get inflamed then it’s easy for us is just to take the patient out and take them to hospital rather than get in to any sort of rows, …. so it can be strained at times,.. it doesn’t happen very often but it does happen occasionally and that’s when it can become very difficult”* [Evidem EoL]*.*
**GP providing an example of when good working relationships mean that she can be confident about a patient, but that this changes if she meets with different staff at every visit:**
*“I find that staff are very experienced, they know their patients (…). So I rely on them a lot to tell me about the changes in behaviour and how they perceive the patient. (…). But if you had a different carer every day, you can’t really make that picture [of the resident’s function]” [Evidem Eol].*
**Care home manager highlighting how family and residents’ wishes shape decision making, and her view that the power to decide is theirs:**
*“[I] do feel that it’s a bit of a fiasco when people decide ‘no, no, I want to still have an intervention’ and it’s chaos towards the end. .... It would be very nice to have a very clear treatment and to have everything crystal clear, but I don’t think that is ever going to happen […] I mean, we can voice an opinion, but we don’t have the right to make those choices (…) we’re proving very much all the time that we’re giving the power to the residents, and that always involves the relatives as well” [EPOCH].*
**Care home staff member that has completed training in EoL care:**
*“In the type of job we do people’s lives are affected, it’s not just the person you are caring for. It’s all of their families….so we have lots of sensitive things to deal with….” [TTT study].*

For ‘out of hours’ services with intermittent contact with the care home, or when there was rapid staff turnover, there were few safeguards to ensure cross organisational working for the resident’s and the family’s benefit. Whilst the use of EoL care frameworks and supporting documentation could support relational working, they were not a surrogate for shared decision making and discussion when there was no on-going review.

At an organisational level there was evidence of uncertainty about how actions and decisions related to EoL care would affect relationships with the regulator and professional bodies respectively. An awareness of the fact that decisions to withhold or stop treatment could be misinterpreted as poor care, particularly in situations when it was unclear if someone was dying, meant that participants felt vulnerable and exposed to challenge. This influenced care home staff confidence to support people with dementia in the care home, decisions not to treat and GPs’ willingness to support residents in the care home. For care home staff, it was the possibility that relatives’ complaints, suggesting care had been withheld, would lead to sanctions by the regulator. For the GP, a decision to withhold medication such as statins could lead to criticism for failing to comply with evidence based practice for prescribing for older people. Whilst irrelevant when someone was actively dying this could be misunderstood if the resident’s trajectory to death was prolonged.

## Service uncertainty

When working relationships were robust, and where the shared use of end of life resources and tools addressed and ameliorated the impact of treatment uncertainty, a care home’s ability to provide EoL care could still be affected by service uncertainty, which is an element of organisational uncertainty. This occurred when the way in which services were organised and resourced informed decisions about who was responsible for continuity of care and where it was provided.

Except in instances where the NHS provided specific time limited funding for EoL life care in the care homes, the costs of keeping a resident in the home reflected assumptions about staff and service availability for someone who needed (predictable) day to day care. In the EVIDEM EoL study, three deaths occurred in the midst of disagreement as to where the resident should be cared for and by whom. This caused prolonged stays in hospital awaiting placement in a care home with nursing. In one case this resulted in a discharge from the hospital back to the care home just one day before death. In the EPOCH study there were examples of residents moving to nursing homes in the last month of life because staffing levels at a residential care home without nursing on site were thought to be insufficient to provide the care needed.

Where the period of dying was protracted, clinicians and care home staff needed to be confident that staff (day and night) had sufficient skills and knowledge to manage symptoms, and that they would be able to access external support as and when needed (see Table 3). Primary care staff were not always confident of their own services’ capacity to provide EoL care support to care homes within existing workloads. Interviews with paramedic staff suggested that decisions about whether or not to take an older person to hospital were influenced by their confidence that the older person would be reviewed by a GP or District nurse within the next 24 h. Similarly, the logistics of working across organisations when resources were in short supply meant that agreements to provide the care home with palliative care support, or with extra equipment such as hospital beds and pressure relieving mattresses, were not always realised. In both the EVIDEM EoL and the EPOCH studies there were examples of hospital beds being delivered to the care home after the person had died.

**Table 3:** Service uncertainty

**Service uncertainty refers to how the organisation and delivery of services can influence decisions about place of care, resource allocation and continuity of care**
**Example of service uncertainty linked to staff changes and the protracted period of dying that overrides good record keeping and shared documentation:**
*“There was somebody who was taken to hospital, … she had dementia and she had end stage kidney failure and heart failure … I’d been treating her for probably* ***three or four years*** *…she started to deteriorate … … it was written in her notes ‘this lady is for palliative care only and is not to be transferred to hospital unless she becomes acutely unwell’…… it was very visible in her summary, in red, unfortunately it was a day that I wasn’t around and she became acutely short of breath and a telephone call was made to the surgery …and it was taken by one of our registrars who just didn’t see the entry on the notes and said ‘call an ambulance’ and she was taken by ambulance to hospital and died that da*y” [EVIDEM EoL].
**Care home manager giving example of where a resident could not be supported in the care home for service reasons:**
*“…, because if I’ve got a ratio of 1 to 8 staff, it’s how much pressure do I put on staff? So, if I’ve got a resident who needs two to three carers, then it’s not actually our criteria…because I can’t up the staff level. It works both ways really, it’s obviously not putting pressure on staff as well as making sure that the person is going to get the best care. [*EPOCH]
*“One of the service level agreements .., is that they agreed with different practices to take a specific interest in certain care homes. That’s been a standing procedure here for quite a while and they [GPs] get some financial redress…. …..but just to have that better communication, so that we all have a better understanding of what we all need, and build these relationships to ensure reliable service provision…”* [Care Home manager-TTT study].

## The development of the uncertainty framework

The analysis of residents’ trajectories of living and dying in care homes, their service use, staff narratives of providing EoL care, and residents’ accounts identified three key linked elements of EoL care that were characterised by uncertainty. The synthesis of the findings from the three studies makes explicit what needs to be simultaneously addressed by EoL interventions to contain uncertainties. In current practice, the focus on the dying trajectory of a person living with dementia is determined by an explicit understanding of how intra and inter-organisational relationships and resources can shape decisions. Many current EoL resources and tools assume that it is possible to identify when someone is close to death and that advance care plans can resolve dilemmas about stopping active treatment and decisions not to admit to hospital. These findings show that while their application may be appropriate for some care home residents with dementia, they are not appropriate for all.

**Fig 1:**
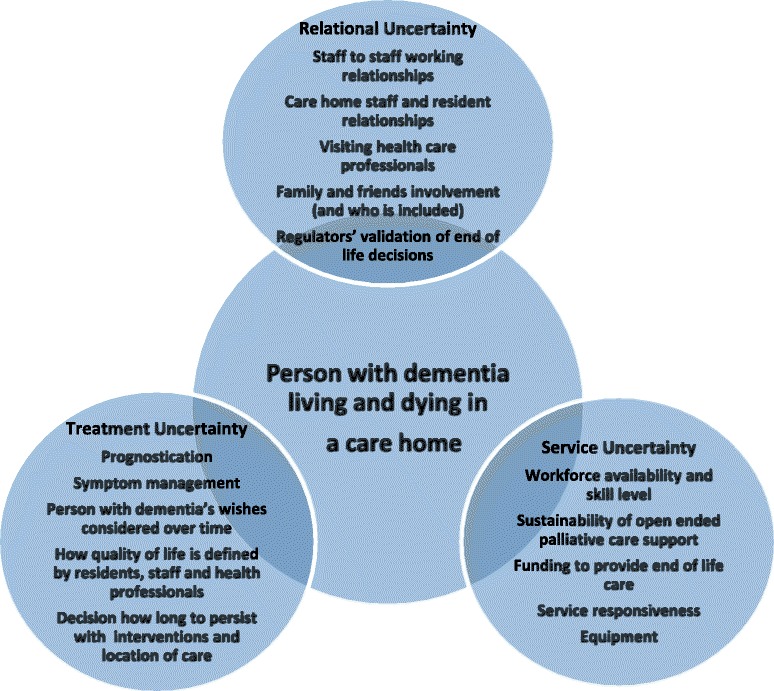
End of life care domains characterised by uncertainty when caring for older people with dementia resident in care homes

The emergent framework (Fig. 1) presents an overview of the issues discussed. It facilitates the mapping of the foci and relative strengths of different EoL interventions for people living with dementia in care homes, and helps to identify where further developmental work or research is required. Interventions that emphasise education and training on symptom management and regular review of residents by visiting professionals and care home staff will be well placed to address symptom management, but may not be as able to address relational uncertainties that arise when it is unclear if someone is actively dying and residents’ relatives disagree with treatment decisions. Equally, even when there is clarity that someone is dying and their preferred priorities for care are known, if primary care services do not have the capacity to provide palliative care support for care home staff, then a different intervention is required. One that can for example, ensure care home staff have advanced palliative care skills, anticipate the need for extra resources or provide intermediary or alternative sources of support such as virtual conferencing or help lines.

## Discussion

This paper has argued that for people with dementia living and dying in care homes uncertainty is an inevitable and integral part of end of life care. Residents’ characteristics, the protracted period of dying, multiple and changing personnel involved, different definitions of quality of life and the realities of working across health and social care mean that uncertainties cannot always be resolved. A situation that is not restricted to the UK setting [9]. It will not always be clear what treatment is the ‘right’ treatment, who should do what and when, and where care should be provided and by whom. We offer an organising framework that incorporates this reality into how EoL care is planned and evaluated for a way forward, to begin the process of comparing the benefits of different EoL care interventions for people with dementia in care homes against one another. It is not surprising for an EoL care intervention to achieve an improvement in the process and outcomes of care, when compared with “usual care” for people with dementia in care homes that are known to have limited access to health care services. The need now is to understand how different interventions address all or some of the uncertainties observed, and whether some are more suited for care home populations than others.

The recent furore and withdrawal of the Liverpool Care Pathway (LCP), in the UK, goes to the heart of assumptions about how dying is recognised, what is known about how people die and who makes the decisions regarding treatment options [28]. There is recognition that even the term “pathway” is misleading with its implications of known direction, shared goals and the ability to standardise care [29]. It is the ability to “hold” uncertainty between the different players and organisations that is the marker of an effective end of life care intervention. It is not enough for dementia specific interventions that include care homes to acknowledge that challenges exist or that they are complex or context sensitive [13, 14, 30, 31].

Instruments that assess quality when dying differs are needed, and few have been developed for long term care settings [32]. Recent reviews and statements about what is needed to evaluate EoL care interventions emphasise a need for an explicit theoretical understanding of how EoL care is defined, and how the effectiveness of different approaches is judged [33, 34].

The emergent framework offers system and organisational awareness, but also unpacks some of the detail of how uncertainty is expressed and experienced in working relationships and decision making at the resident and service level of care. Indeed, acknowledging relational uncertainties that arise from multiple people being involved, shifting responsibilities and the need to maintain relationships over time allows for a clearer understanding of how service and trajectory uncertainties can be managed. For people with dementia in care homes, understanding how dementia complicates dying is not enough. There is also a need for interventions that can create trust and patterns of working and communicating that cross organisational boundaries, build in service flexibility at times of crisis and/or when the commitment required is open ended.

## Strengths and weaknesses

The analysis presented in this paper draws on data from three discrete but complementary studies on end of life care for people living and dying in care homes. The data on care received and services used by 528 residents were directly comparable across the three studies. Similarly, the interviews with 205 care home staff, and 44 visiting health care professionals all included prompts that addressed the experience of giving and receiving end of life care for care home residents. A concern about secondary analysis of qualitative data is the extent to which context, and the knowledge that a researcher has through ‘being there’ in the research setting can be adequately summarized and captured for use by anyone other than the primary researcher [27]. It is a particular strength that the authors were direcetly involved in the included studies.

This framework has particular relevance for care homes that rely on visiting health care professionals for palliative care. Further comparative work is needed to test its relevance for non UK settings, particularly those with on-site physicians. It is a weakness that only one study exclusively included people with dementia however; in the UK the overwhelming majority of care home residents have a degree of cognitive impairment.

## Conclusion

End of life interventions for people with dementia in care homes need to demonstrate effectiveness in their capacity to manage or “hold” the inherent uncertainty of living and dying with dementia in long term residential care settings. The cumulative findings from the three studies demonstrate that there is a need to understand better how these three aspects or key elements of end of life differentially affect decision making and the experience of living and dying in long term care for people with dementia and what needs to be addressed to achieve a level of “practical certainty” that is workable and not just aspirational.

### Availability of data and materials

Not Applicable.

## Abbreviations

ACP: Advance care plan
EoL: End of life
EVIDEM EoL: “Evidence-based Interventions in Dementia: Changing practice in dementia care in the community: developing and testing interventions from early recognition to end of life”
EPOCH: Experiences of older people in care homes
GP: General practitioners
LCP: Liverpool care pathway
TTT EoL: Train the trainer: an end of life care education and training programme
UK: United Kingdom
NHS: National health service
NIHR: National institute for health research
